# Supplementary material for: Platelet activation near point-like source of agonist: Experimental insights and computational model
Source: PLoS One. 2024 Oct 3;19(10):e0308679. doi: 10.1371/journal.pone.0308679 (PMC11449293; doi:10.1371/journal.pone.0308679)
Supplement: S1 File — (PDF) [file pone.0308679.s001.pdf]

Platelet activation near point-like source of agonist: theoretical model and experimental verification

Ezhena S. Starodubtseva[1], Tatyana Yu. Karogodina[1,2], Alexander E. Moskalensky[1]

[1] Novosibirsk State University, Novosibirsk, Russia
[2] N.N. Vorozhtsov Novosibirsk Institute of Organic Chemistry SB RAS, Novosibirsk, Russia

Table S1. The parameters of the delay time TD for N = 3 donors

Donor, №	μ_D	σ_D	Median [TD]	Mode [TD]
1(A, B)	1.97	1	7.17	2.63
2 (C, D)	2.02	0.64	7.54	5
3(E, F)	1.89	0.87	6.62	3.1

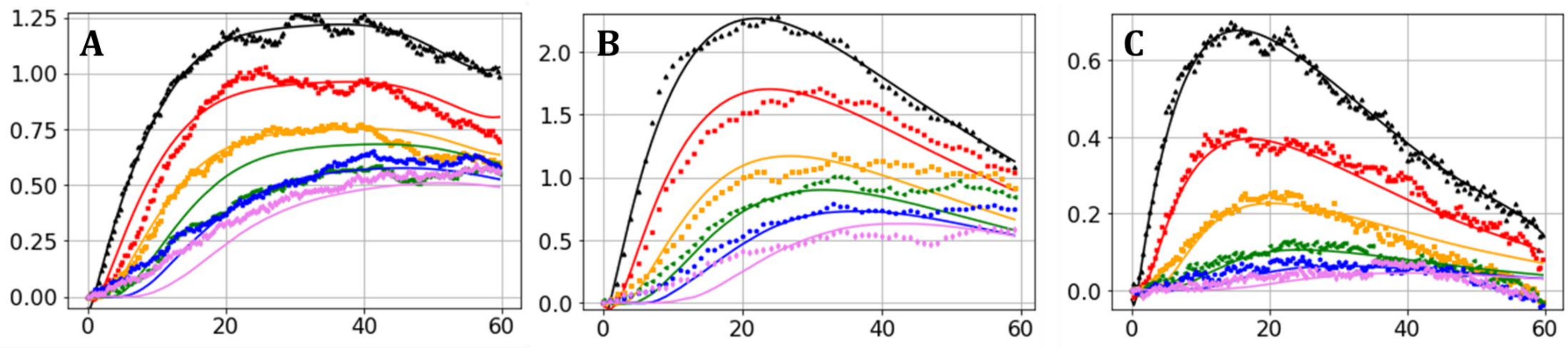

Fig. S1. Overlayed experimental and theoretical data. A. Corresponding parameters are represented in Table S1

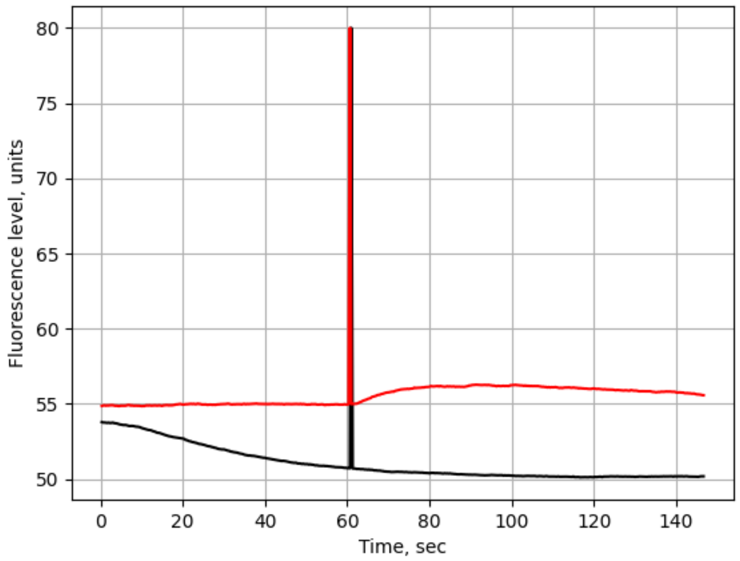

Fig. S2. Comparison of control experiment (black) without addition of caged ADP and experiment with caged ADP (red). A spike represents the appearance of laser flash. Control shows that laser does not have any influence on the sample itself and without agonist in the system the signal is slowly decreasing. On the other hand, the presence of caged ADP does not influence the system before its excitation and makes the signal rapidly increase after the «uncaging» of the agonist.

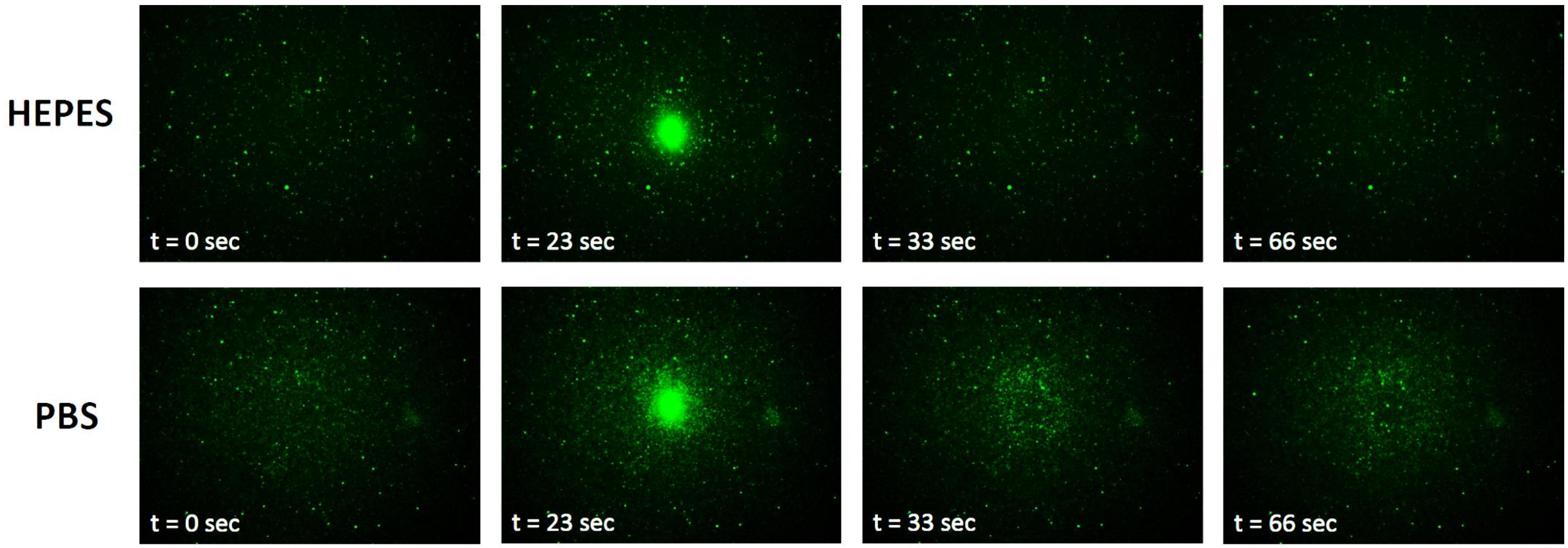

Fig. S3. Comparison of buffers used in experiments. Upper row: HEPES buffer; lower row: PBS. In the experimental conditions, platelet activation after laser flash (which is showed in 2nd column) was observed in PBS only was not observed in HEPES buffer.
