# Supplementary figures and images for: Platelet activation near point-like source of agonist: Experimental insights and computational model
Source: PLoS One. 2024 Oct 3;19(10):e0308679. doi: 10.1371/journal.pone.0308679 (PMC11449293; doi:10.1371/journal.pone.0308679)

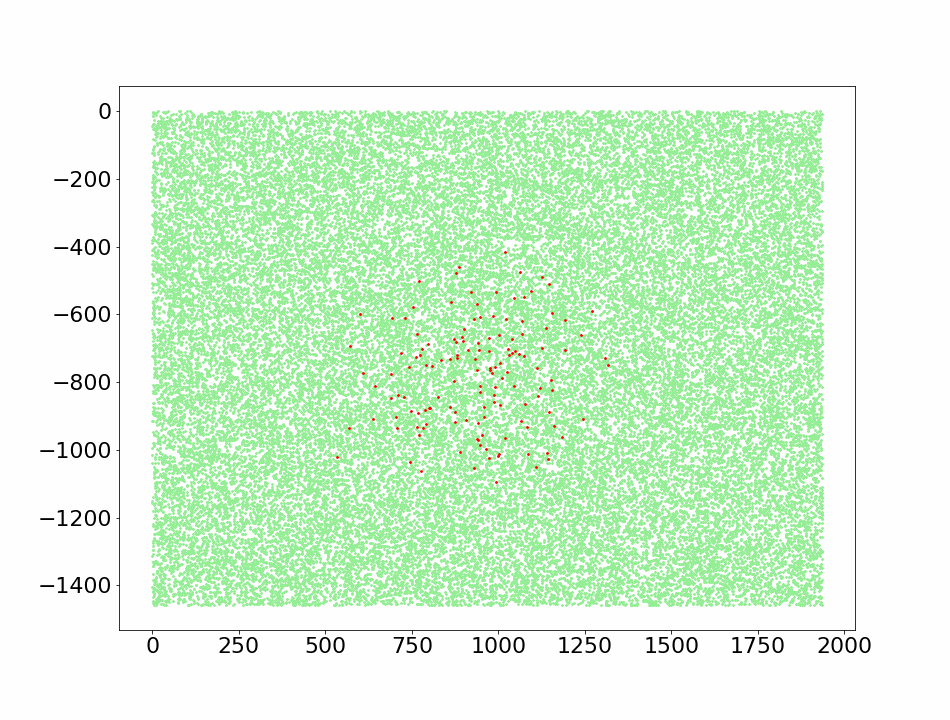

Supplement: S2 Data — (GIF) [file pone.0308679.s004.gif]
