# Supplementary material for: Platelet activation near point-like source of agonist: Experimental insights and computational model
Source: PLoS One. 2024 Oct 3;19(10):e0308679. doi: 10.1371/journal.pone.0308679 (PMC11449293; doi:10.1371/journal.pone.0308679)
Supplement: Attachment — Submitted filename: PLOS_reviewers Ezh.pdf [file pone.0308679.s005.pdf]

We thank the reviewers for their constructive comments. In fact, we have re-done experiments with better temporal resolution and extended the number of healthy volunteers to N = 3. Novel data are presented in the paper and Supplementary materials. Below is point-by-point answer to the reviewers.

Reviewer #1: The work of Starodubtseva et al. is devoted to the development of the combined theoretical and experimental approach for the evaluation of intracellular calcium signaling responses in human platelets. Using UV-uncagable ADP the authors were able to trigger localized ADP concentration changes that would induce calcium oscillation in the adjacent platelets. In line with their previous works, they have constructed a theoretical framework to derive platelet calcium response parameters that could be of importance for the evaluation of the platelet condition. Despite the overall elegance of the experimental setup and the complementary computational approach, there is a set of flaws that do not allow this work to be published in the current state.

Major points:

The authors claim that their approach can be of clinical significance since the derived theoretical parameters can be used to characterize platelets. However, according to the methods section, there is only one healthy donor included in the study. Therefore, it is impossible to determine the potential variability of the derived parameters. The N of healthy donors should be increased by at least N=3.

We have conducted additional experiments to increase the number of healthy volunteers in this study. Overall, the blood samples of three healthy donors (female 22yo, male 35yo and female 40yo) were analyzed. We changed the description in methods section accordingly.

A set of concerns about the experimental approach should be addressed:

In https://doi.org/10.1080/09537104.2023.2229909 authors have demonstrated that citrate affects platelet functioning, namely, inhibits platelet responses. Therefore, it can be expected that to preserve platelet function, the authors should reduce platelet exposure to citrate to a minimum. However, instead, the authors leave the whole blood for 1.5h at room temperature to obtain platelet-rich plasma. If the authors claim that their approach is more efficient than platelet centrifugation, they should provide corresponding data.

The samples were collected according to the standard protocol to the vacuum tubes containing sodium citrate. We tried to keep the protocol as simple as possible and reduce a number of steps to avoid unnecessary influence on platelets. We agree that prolonged exposure to citrate may affect platelet reactivity, but in our protocol the platelet function is preserved enough to obtain the investigated effect experimentally.

In the future, optimization of protocol may provide much better results. However, as we did not study the specific effect of centrifugation, for now we removed the claim “In order to avoid spontaneous mechanical activation and ensure that the majority of platelets will keep their initial state, the centrifugation was not used”, leaving only “Centrifugation was not used”.

How do authors ensure that they observe single platelets instead of platelet aggregates, stacked upon each other, since they make observations using a low magnification microscope?

The image capture was performed using 10x magnification objective lens. Single cells at this magnification are visible as bright dots comprising several pixels. Aggregates (e.g. two adjacent cells) can be distinguished from single cells even at this magnification, and we did not observe them in the described experiments. Also, the majority of cells are freely moving, which is visible due to Brownian motion. It means that they are not adherent to a surface or spread platelets (which are also present).

The authors should specify the coating of the glass that was used for platelet immobilization before microscopy analysis.

The surface was made of polystyrene. We have indicated this in the Sample preparation section.

The images of the platelet calcium responses the authors provide in Fig. 3 differ from what is typically obtained in single platelet microscopy (https://www.sciencedirect.com/science/article/pii/S1538783622035607), presumably due to lower temporal resolution. Therefore, platelet calcium concentration profiles with higher temporal resolution should also be provided.

We thank the reviewer for this suggestion. Indeed, the initial experiment was performed with the frame rate of 1 f/s, which is quite small. Following the reviewer's request, we have conducted new experiments with better temporal resolution of 3 f/s. The resulting calcium spikes are indeed more similar to classical results. In addition, results shown in Figure 4 is now much better than before.

We have modified the description of Figure 3 in the text and added reference that the reviewer pointed out:

"Rapid spikes with further decrease indicate the activation response, as observed in classical studies [13,30]"

ADP-induced calcium activation occurs almost momentarily (https://journals.plos.org/plosone/article?id=10.1371/journal.pone.0122527), while here authors claim that the spikes occur 10-15 seconds after the uncaging of the ADP. The authors should address this.

Experimental results in the present paper and our previous study https://doi.org/10.1364/BOE.392745 indicate that spikes occur with some delay. However, it may depend on the strength of activation (concentration of ADP). In the paper pointed out by the reviewer activation indeed occurs very fast according to Figure 5:

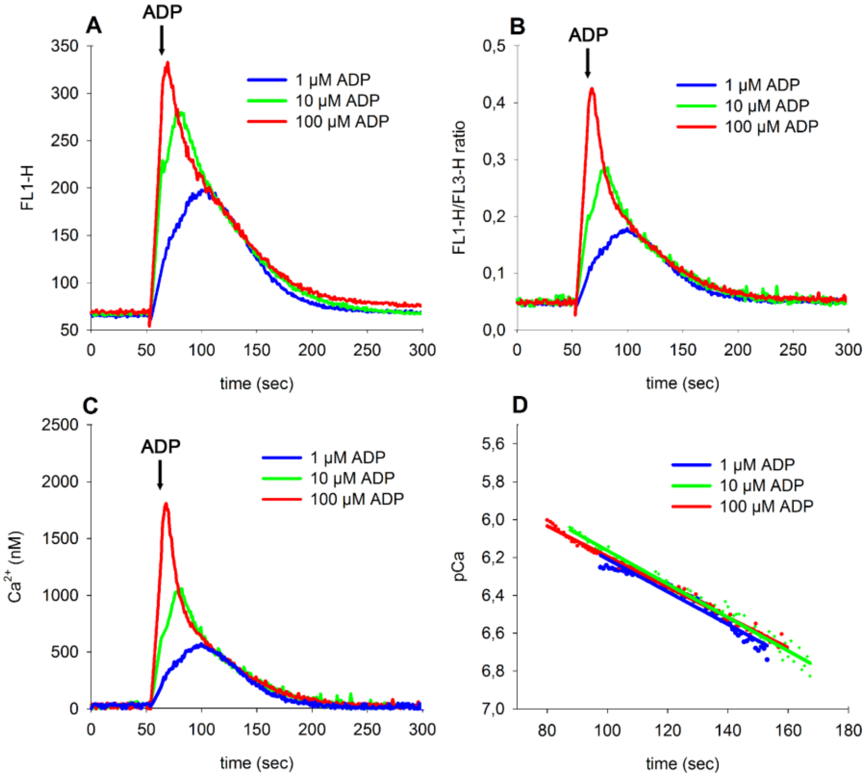

However, when we tried to estimate the delay time from this figure, we found that the fluorescence growth offset is actually earlier than the indicated moment of ADP addition. Therefore, we believe that this moment was shown approximately and it is impossible to evaluate the delay time from the paper:

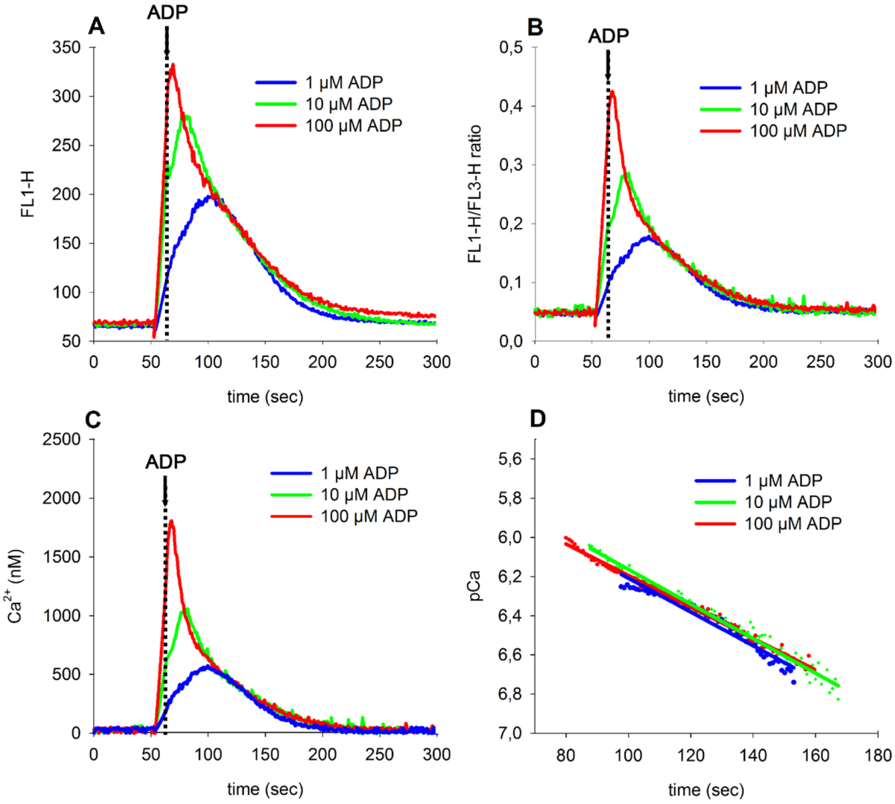

On the other hand, delayed activation was demonstrated e.g. in https://doi.org/10.1083/jcb.201306085 where the authors studied marginal band coiling in platelets and showed that it occurs minutes after the addition of ADP.

> In their theoretical framework, the authors often refer to their previous works (references 26, 27, 28), specifically in the determination of the platelet activation parameters. However, it is not obvious, how platelet shape change (MPSI) and platelet sensitivity distribution width (PSDW) correlate with platelet calcium signaling. The authors should at least prove that the processes have linear dependence or justify the application of these parameters in some other way.

Indeed, the platelet morphological parameters such as the shape change distribution and its derivatives have complex relation to the activation. Ref. 27 was indeed misplaced and we removed it. In ref. 28, we studied platelet activation using the same approach as in the present paper (activating with caged ADP and measuring calcium spikes). The most confusing is Ref. 26 where the platelet sensitivity threshold to ADP, designated as MPS-ADP, was estimated by measuring the shape change. It is MPS-ADP that we refer to in the present paper. This parameter is directly related with the concentration of ADP required to trigger the shape change, and therefore can be compared to the threshold concentration of ADP obtained in our present paper.

The MPSI-x parameters from Ref. 26 are indeed related to the shape distribution and not ADP concentration, but unfortunately have confusingly similar abbreviation. We modified the sentence in discussion to avoid confusion: The obtained sensitivity parameters, namely the mode of distribution that is near 10^{-7} M, are in range of the previously published values, in particular, the platelet sensitivity parameter MPS-ADP which was determined in [26] by measuring the shape change.

> In Fig. 4 authors demonstrate the comparison of the theoretical (B) and the experimental (C) distributions of activated platelets for different regions. Despite what they claim in the corresponding manuscript section, the distributions do not seem to be “in qualitative agreement”. The authors should overlay the distributions and should also explain why in sections 1 and 2 in the experimental T to activation is longer than the theoretical one.

This figure was modified due to three reasons. First, novel experimental data with better time resolution allows more reliable determination of single calcium spikes. It leads to more smooth experimental profiles. Second, we have corrected an error which was in a fitting function (lognormal probability density) in the previous version of the paper. Now the histograms are indeed in a qualitative agreement, i.e. have comparable means and widths. Third, all histograms are now normalized (probability density is shown).

Still, the agreement is not ideal and we decided not to overlay them because the purpose of Fig. 4 is to show that the theory captures main features of experiment (activation spread) but cannot accurately describe other features. For instance, by comparing D and E panels, one can see activation of cells in the most distant areas from the start of experiment, whereas theory predicts certain lag-time before the activation could start in these regions. We have modified the sentence as follows: “Although the overall results are in qualitative agreement, simulation predicts the lag-time in distant areas, which is absent in experiment, probably indicating the presence of extra-sensitive platelets.”

The authors first obtain the distribution parameters for individual platelet profiles (μD=1.4 and σ=0.9) and then, I assume, try to generalize their method for the larger areas. Therefore, one would assume that the log-normal distribution parameters for the areal model would provide similar results, however, the obtained values significantly differed.

The parameters μD and σD refer to a distribution of delay time between stimulus and activation. They are determined for all platelets at once and do not depend on coordinates or area. Later these parameters are used in an analysis of time distribution of calcium spikes presented in the Fig. 4.

Another concern for this approach is that the authors "consider each cell to contribute a smooth growth-and-decay function to a total signal", while the smooth calcium signal (typically observed for the platelets for example in FACS) upon platelet activation by ADP is a superposition of the calcium oscillations of platelets in the suspension. Therefore in the current form, the authors' generalization does not appear legitimate.

In the analysis of data presented in the Fig.5 we use only mean fluorescence signals from rings which contain a lot of platelets. In this case we do not use the distribution of delay time, instead we take experimental signal from the ring nearest to the laser flash and then modify it according to the diffusion model described in the theory section to simulate signals in other areas. This approach uses less assumptions than that in Fig.4, which explain better agreement with experimental data in Fig. 5. We have added the sentence: "This is justified because there are many cells in the areas under consideration and all spikes are well averaged; in other words, each "cell" in the simulation can be considered as an ensemble of many cells.".

Minor points:

Scale bars in Fig. 3 are barely visible. In Fig. 3E-G the letters overlap with the experimental profiles. The dot size on the plots should be decreased or dots should be just removed and only the curves left, since the dots obscure the experimental curve patterns.

Fixed.

In Fig. 4A there is no "0 sec" surve, although there is a legend for it.

Fixed.

It is better to overlay experimental and theoretical data in order to better demonstrate the accuracy of the fit.

We tried this, but the resulting plots were hard to understand, so we leave the same figure structure in the main text (only extended it for 3 donors). But we have added the overlay to supplementary file.

The authors should demonstrate the experimental regions for the data in Fig. 4.

This was fixed.

Reviewer #2: The authors present a study of spatial-temporal platelet activation by ADP. They use an interesting experimental approach whereby "caged" ADP is released from the platelets after laser stimulation. The ADP then triggers platelet activation, monitored through calcium signals, and diffuses to nearby platelets to activate them, and so forth.

Experimental data tracks the fraction of activated platelets in space and time and a very simple model based on diffusion is used for the ADP, these simulated dynamic concentrations are then used to estimate the activation of platelets in space and time. The authors conclude that they developed a novel model that was able to predict threshold-like platelet activation responses and further suggest that their approach could be used in larger-scale models of thrombus growth. It was my overall impression that more work is needed to substantiate the conclusions of novelty and applicability in the manuscript. It is known that ADP diffuses and activates platelets with a threshold value (see Zhou & Schmaier 2005), so it is unclear how this work contributes new biological insights. For these reasons, I do not think this work is suitable for PLOS Computational Biology.

The fact that ADP activates platelets is indeed well-known, as well as the fact that ADP is able to diffuse in liquid medium. Our paper brings these two facts together by making inhomogeneous ADP distribution and tracking the time-dependent response of the system. This is quite novel experimental approach, at least in platelet research, which allows one to answer the following questions:

1. How different are platelets in terms of ADP sensitivity threshold, and can their distribution over this threshold be considered log-normal?
2. Do the platelets exhibit traveling-wave behavior, characteristic to systems with positive feedback mechanisms (i.e. can the activation waves traveling from the activation point be formed due to secondary activation, granule release etc?).
3. What is the sensitivity threshold to ADP for platelets of the particular donor/patient? What is the distribution width? (can be determined after one simple measurement).

Overall, even if our experimental study confirms known concepts, the presented approach can be used in the future to study advanced activation stimuli, as we pointed out in the last paragraph of the discussion.

Finally, as PLOS ONE accepts scientifically rigorous research, regardless of novelty, we hope that additional work to substantiate novelty and applicability can be leaved for our future papers.
